# Evaluation of candidate genes associated with hepatitis A and E virus infection in Chinese Han population

**DOI:** 10.1186/s12985-018-0962-2

**Published:** 2018-03-20

**Authors:** Maolin Gu, Jing Qiu, Daoxia Guo, Yunfang Xu, Xingxiang Liu, Chong Shen, Chen Dong

**Affiliations:** 1 Huai’an Forth Hospital, Huai’an, China; 2 Department of Epidemiology and Statistics, School of Public Health, Jiangsu Key Laboratory and Translational Medicine for Geriatric Disease, Medical College of Soochow University, 199 Renai Road, Suzhou, 215123 China; 3 Department of Epidemiology, School of Public Health, Nanjing Medical University, 101 Longmian Road, Nanjing, 211166 China

**Keywords:** Hepatitis A virus, Hepatitis E virus, Single-nucleotide polymorphism

## Abstract

**Background:**

Recent GWAS-associated studies reported that single nucleotide polymorphisms (SNPs) in ABCB1, TGFβ1, XRCC1 genes were associated with hepatitis A virus (HAV) infection, and variants of APOA4 and APOE genes were associated with and hepatitis E virus (HEV) infection in US population. However, the associations of these loci with HAV or HEV infection in Chinese Han population remain unclear.

**Methods:**

A total of 3082 Chinese Han persons were included in this study. Anti-HAV IgG and anti-HEV IgG were detected by enzyme-linked immunosorbent assay (ELISA). Genotypes in ABCB1, TGFβ1, XRCC1, APOA4 and APOE SNPs were determined by TaqMan MGB technology.

**Results:**

In Chinese Han population, rs1045642 C to T variation in ABCB1 was significantly associated with the decreased risk of HAV infection (*P* < 0.05). However, the effect direction was different with the previous US study. Rs1001581 A to G variation in XRCC1, which was not identified in US population, was significantly associated with the protection against HAV infection in our samples (*P* < 0.05). In addition, our results suggested that rs7412 C to T variation in APOE was significantly associated with lower risk of HEV infection in males (adjusted OR < 1.0, P < 0.05) but not in females.

**Conclusions:**

ABCB1 and XRCC1 genes variants are significantly associated with the protection against HAV infection. Additionally, Chinese Han males with rs7412 C to T variation in APOE gene are less prone to be infected by HEV.

**Electronic supplementary material:**

The online version of this article (10.1186/s12985-018-0962-2) contains supplementary material, which is available to authorized users.

## Background

Hepatitis A and E virus (HAV and HEV) infections are heavy public health problems in developed and developing countries. It is estimated that over ten millions people have been infected by these two viruses every year worldwide [1, 2]. Both HAV and HEV are mainly transmitted via the fecal-oral route either by the contaminated food or water ingestion or via person-to-person contact. Although the disease of hepatitis A or hepatitis E is usually mild or asymptomatic, the higher incidences of fulminant hepatitis could be occurred in the HAV-infected elders and HEV-infected women in pregnant [3, 4].

In the past decade, Genome-wide association studies (GWAS) have been carried out rapidly and contributed to identify susceptible loci for many kinds of infectious diseases, such as malaria, tuberculosis, hepatitis B and C [5–7]. In a US study, Zhang et al. recently reported that Mexican Americans carrying the functional T allele of TGFβ1 rs1800469 and T allele of XRCC1 rs1799782 were more prone to be infected by HAV, while the minor allele (T) of ABCB1 rs1045642 was associated with lower risk for anti-HAV seropositivity [8]. TGF-β1 is a multifunctional cytokine and involved in the pathogenesis of liver injury during acute HAV infection. Moreover, it has been reported that TT genotype of TGFB1 rs1800469 is significantly associated with higher plasma levels of TGF-β1 [9]. XRCC1 is a major DNA repair gene so it has ability to repair DNA damage, which may be a requisite for risk of many infectious diseases [10]. Recently, the results from a 10-year cohort study indicated that XRCC1 gene may be responsible for hepatocellular transformation in Chinese chronic hepatitis B patients [11]. ABCB1 gene encodes one member of the ATP-binding cassette super family of membrane transporters. Up-regulated expression of ABCB1 in human hepatocytes in viral hepatitis may render the cells resistant to oxidative damage [12].

In another study, Zhang et al. further reported that rs5110-C/A in APOA4 and rs7412-C/T in APOE were significantly associated with protection against HEV infection in non-Hispanic blacks in United States [13]. Both APOA4 and APOE genes were recognized for their importance in lipoprotein metabolism and cardiovascular disease. However, more and more studies indicated that these two genes are not only play the key roles in lipid transport, but also involved in immunoregulation, which in turn can influence susceptibility to and the natural history of infectious disease [14, 15].

However, the associations of these loci with HAV or HEV infections have not been evaluated in other populations. Owing to its long history, large population, environmental pollution, and poor hygiene, both hepatitis A and E outbreaks and sporadic incidents are the major public health problems in China [16, 17]. Additionally, accumulative evidences demonstrated that the seroprevalence of HAV and HEV infections are very high in Han ethnic areas (~ 60% and ~ 20% for anti-HAV IgG and anti-HEV IgG, respectively) [16]. Given the additional evidences of genetic associations will improve our understanding of genetic factors and mechanisms truly influencing HAV or HEV infection, we performed the present study to evaluate the single-nucleotide polymorphisms (SNPs) recently reported to be associated with HAV or HEV infection in Chinese Han population.

## Methods

### Study participants and data collection

This study was performed with 3082 Chinese Han persons from the Huai’an-09 Study, which is established in 2009 and is designed to investigate the health conditions of general populations in Huaian, China. Between April 2009 and September 2009, 3166 individuals (aged 30–75 yrs) were randomly selected from Huai’an city. All participants underwent questionnaire assessment, physical examination, and routine blood, urine, and biochemical tests at baseline without charge. All the doctors and nurses had rigorous unified training before they measured the anthropometric indices. Blood samples were obtained by venipuncture and stored at − 80 °C until further analysis.

In the current study, we excluded 84 persons including 83 individuals with HBsAg positive, one person with anti-HCV IgG positive. None of participants tested for antibodies to human immunodeficiency virus. In addition, people with hepatic fibrosis, diabetes, renal inadequacy and cancer were excluded. Eventually, 3082 persons were finally included in this study.

### Laboratory testing

Both anti-HAV IgG and anti-HEV IgG were detected by enzyme-linked immunosorbent assay (ELISA) with commercial reagents purchased from Wantai Bio, Beijing, China. According to the manufacturer’s instructions, the sensitivity and specificity is 98% and 97% for the anti-HAV IgG detection, and 99% and 97% for anti-HEV IgG detection, respectively.

### SNP selection and genotyping

There were five SNPs have been reported to be associated with HAV infection in US GWAS studies [8]. Because the minor allele frequency (MAF) of rs1799782 in XRCC1 was lower than 5% in Han Chinese, we analyzed the associations of rs1001581in XRCC1 (MAF: 0.44; LD: r^2^ > 0.8 with rs1799782 in Caucasian population) with HAV infections in the present study. Additionally, the genotypes of rs1045642 in ABCB1, rs1800469 in TGFB1, and two SNPs marginally associated with anti-HAV (rs2031920 in CYP2E1 and rs769214 in CAT) were selected to evaluation according to the findings of Zhang’s study [8].

Rs5110 in APOA4 and rs7412 in APOE gene were reported to be associated with HEV infection in Zhang’s study. However, MAF of rs5110 in Chinese population (HapMap) was much lower (MAF = 0 for CHB and MAF = 0.023 for HCB). We selected rs1268354 in the present study because rs1268354 is the only polymorphism with MAF > 0.05 in APOA4 in Chinese population (MAF = 0.45). In addition, Zhang et al. suggested that there was a significant joint effect of rs7412 and rs429358 on HEV infection [13]. Considering MAF of rs429358 was not available in Chinese population and rs429358 had lower LD (r^2^ < 0.1) with rs405509 or rs769450 in Caucasian population, rs7412, rs405509 (MAF: 0.31) and rs769450 (MAF: 0.20) were selected to analysis in our present study.

Proteinase K digestion and phenol-chloroform extraction were used to isolate genomic DNA from EDTA-treated blood samples. Genotyping was performed using TaqMan MGB technology and 7900HT Fast Real-Time PCR System (Applied BioSystems, Foster City, CA, USA). The commercial reagents including primers and probes were ordred from Applied BioSystems and Biosteed Biotechnologies (Nanjing, China), respectively (Additional file 1: Table S1). According to the manufacture, the results from TaqMan assay used in the present study are in a concordance of 99.8–99.9% with the results from sequencing. The genotypes were determined automatically with the Sequence Detection System 2.1 software (95% autocaller confidence level). In the present study, 5% samples were duplicated and the results were in a concordance of 100%. The genotype-calling success rates were greater than 99.9%.

### Statistical analysis

Fisher’s exact test was used to test for Hardy-Weinberg equilibrium (HWE). The genotype and allele distribution were analyzed using chi-squared (χ^2^) tests. The odds ratio (OR) and 95% confidence intervals (CI) were calculated by logistic regression (adjusted for age and gender) and used to test for the risk of associations. All of the statistical analyses were performed with SPSS version 17.0 (SPSS, Inc., Chicago, USA). A two-tailed *P* < 0.05 was considered statistically significant.

## Results

### Characteristics of studied population

Totally, 1655 males and 1427 females were included in the present study, the mean age of males and females were 44.84 ± 8.45 and 44.58 ± 8.50 years, respectively. No significant difference was observed between males and females (Table 1).

**Table 1:** Characteristic of HAV IgG and HEV IgG antibody in studied population

Characteristic	HAV IgG				HEV IgG			
	Positive (*n* = 2375)	Negative(*n* = 707)	χ^2^/*t*	*P*-value	Positive (*n* = 885)	Negative(*n* = 2194)	χ^2^/*t*	P-value
Age (Years)	45.33 ± 8.35	42.66 ± 8.76	7.376	<0.001	45.89 ± 8.53	44.24 ± 8.47	4.884	<0.001
30-	519(65.5%)	2327(34.5%)			174(22.0%)	618(70.8%)		
40-	1284(80.6%)	310(19.4%)			473(29.7%)	1120(70.3%)		
50-	427(83.1%)	87(16.9%)	88.838	<0.001	171(33.4%)	341(66.6%)	29.649	<0.001
60-	127(79.4%)	33(20.6%)			59(36.9%)	101(63.1%)		
70-	18(81.8%)	4(18.2%)			8(36.4%)	14(63.6%)		
Gender								
Male	1299(54.7%)	356(50.4%)	4.130	0.042	486(54.9%)	1166(53.1%)	0.795	0.373
Female	1076(45.3%)	(35,149.6%)			399(45.1%)	1028(46.9%)		

We firstly analyzed the seropositivity of anti-HAV IgG in the Chinese Han population. As the results shown, 2377 participants were determined with anti-HAV IgG positive (77.06%). Among of them, 1299 males (78.49%) were anti-HAV IgG positive, which was significantly higher than those in the females (75.40%, χ^2^ = 4.130, *P* = 0.042, Table 1).

Next, anti-HEV IgG were measured in the studied population. Three samples were not measured because of samples insufficient. As the results shown, the seroprevalence of anti-HEV IgG was 28.72%. Four hundred eighty-six males (29.37%) and 399 females (27.96%) were positive for anti-HEV IgG, respectively. There was not significant deference between males and females (χ^2^ = 0.795, *P* = 0.373, Table 1).

### Genetic associations with anti-HAV IgG seropositivity

There were no significant deviations from HWE for the selected polymorphisms (Additional file 2: Table S2). As presented in Table 2, rs1045642 C to T variation in ABCB1 and rs1001581 A to G variation in XRCC1 gene were significantly associated with the decreased risk of HAV infection (OR < 1.0 adjusted for gender and age) in Chinese Han population. However, the remaining 3 SNPs, rs769214 in CAT, rs1800469 in TGFB1 and rs2031920 in CYP2E1, which were shown to be associated with HAV infection in Mexican Americans, were not associated with anti-HAV seropositivity in our samples (*P* > 0.05, adjusted for gender and age; Table 2). In subsequent gender-differentiated analyses, we did not observe any association of these five SNPs with HAV infection (Table 3).

**Table 2:** Analysis on the association between SNPs selected from GWAS and anti-HAV IgG seropositivity

Gene nomenclature	SNP	Anti-HAV IgG	WT/HT/MT	OR(95%CI)^a^		
				Additive	Dominant	Recessive
CAT	rs769214		GG/GA/AA			
		Positive	1190/975/201	0.979(0.858–1.117)	0.966(0.815–1.144)	1.000(0.738–1.355)
		Negative	347/298/60	*P* = 0.754	*P* = 0.687	*P* = 0.999
XRCC1	rs1001581		AA/AG/GG			
		Positive	929/1040/403	0.920(0.817–1.036)	0.836(0.700–0.998)	0.995(0.794–1.247)
		Negative	247/338/120	*P* = 0.168	*P* = 0.047	*P* = 0.966
ABCB1	rs1045642		CC/CT/TT			
		Positive	913/1107/346	0.862(0.763–0.974)	0.835(0.699–0.997)	0.802(0.638–1.007)
		Negative	245/334/123	*P* = 0.017	*P* = 0.047	*P* = 0.057
TGFβ1	rs1800469		CC/CT/TT			
		Positive	600/1194/572	0.918(0.813–1.037)	0.878(0.719–1.073)	0.908(0.747–1.104)
		Negative	162/362/181	*P* = 0.169	*P* = 0.204	*P* = 0.332
CYP2E1	rs2031920		CC/CT/TT			
		Positive	1471/780/113	0.868(0.754–1.001)	0.851(0.716–1.012)	0.799(0.551–1.159)
		Negative	409/254/41	*P* = 0.051	*P* = 0.068	*P* = 0.237

*WT* wild type, *HT* heterozygote, *MT* mutant type

^a^: Adjusted for age and sex

**Table 3:** Gender-stratification analysis on the association between selected SNPs and anti-HAV seropositivity

Gene nomenclature	SNP	Stratum	Anti-HAV IgG	WT/HT/MT	OR(95%CI)/*P* value^a^		
					Additive	Dominant	Recessive
CAT	rs769214			GG/GA/AA			
		Male	positive	654/525/115	0.955(0.797–1.144)	0.920(0.727–1.163)	1.019(0.673–1.544)
			negative	172/152/31	*P* = 0.618	*P* = 0.485	*P* = 0.928
		Female	positive	536/450/86	0.994(0.822–1.201)	1.000(0.786–1.273)	0.965(0.622–1.498)
			negative	175/146/29	*P* = 0.946	*P* = 1.000	*P* = 0.875
XRCC1	rs1001581			AA/AG/GG			
		Male	positive	491/577/229	0.911(0.774–1.073)	0.824(0.644–1.055)	0.978(0.720–1.328)
			negative	119/173/64	*P* = 0.266	*P* = 0.125	*P* = 0.888
		Female	positive	438/463/174	0.925(0.781–1.096)	0.842(0.656–1.081)	1.010(0.727–1.404)
			negative	128/165/56	*P* = 0.369	*P* = 0.177	*P* = 0.951
ABCB1	rs1045642			CC/CT/TT			
		Male	positive	497/624/174	0.848(0.714–1.007)	0.827(0.646–1.059)	0.773(0.561–1.067)
			negative	120/174/59	*P* = 0.061	*P* = 0.131	*P* = 0.117
		Female	positive	416/483/172	0.900(0.759–1.067)	0.879(0.684–1.129)	0.852(0.621–1.169)
			negative	125/160/64	*P* = 0.224	*P* = 0.312	*P* = 0.321
TGFβ1	rs1800469			CC/CT/TT			
		Male	positive	350/637/307	0.910(0.771–1.074)	0.820(0.623–1.079)	0.947(0.721–1.244)
			negative	83/185/88	*P* = 0.265	*P* = 0.156	*P* = 0.697
		Female	positive	250/557/265	0.947(0.796–1.127)	0.962(0.721–1.283)	0.904(0.687–1.190)
			negative	79/177/93	*P* = 0.542	*P* = 0.792	*P* = 0.471
CYP2E1	rs2031920			CC/CT/TT			
		Male	positive	795/438/61	0.865(0.712–1.051)	0.868(0.684–1.102)	0.714(0.435–1.171)
			negative	206/126/23	*P* = 0.144	*P* = 0.244	*P* = 0.180
		Female	positive	676/342/52	0.860(0.702–1.052)	0.810(0.633–1.037)	0.938(0.542–1.628)
			negative	203/128/18	*P* = 0.142	*P* = 0.094	*P* = 0.823

*WT* wild type, *HT* heterozygote, *MT* mutant type

^a^ Adjusted for age

### Genetic associations with anti-HEV IgG seropositivity

Table 4 summarized the associations between genetic variants in APOA4 and APOE genes and the risk of HEV infection. Rs7412-C in APOE, which was identified in American non-Hispanic blacks, was not associated with seropositivity of anti-HEV IgG in Chinese Han population (P > 0.05, adjusted for gender and age). Similarly, the remaining three SNPs, rs1268534 in APOA4, rs405509 and rs769480 in APOE did not show any relationships with anti-HEV seropositivity.

**Table 4:** Analysis on the associations between SNPs selected from GWAS and anti-HEV IgG seropositivity

Gene nomenclature	SNP	Anti-HEV IgG	WT/HT/MT	OR(95%CI)^a^		
				Additive	Dominant	Recessive
APOA4	rs1268354		CC/CT/TT			
		Positive	341/413/129	0.929(0.828–1.043)	0.88(0.748–1.034)	0.967(0.775–1.208)
		Negative	778/1083/324	*P* = 0.212	*P* = 0.121	*P* = 0.77
APOE	rs405509		TT/TG/GG			
		Positive	443/377/62	0.996(0.88–1.127)	1.035(0.884–1.211)	0.863(0.639–1.166)
		Negative	1120/886/182	*P* = 0.944	*P* = 0.67	*P* = 0.337
	rs7412		CC/CT/TT			
		Positive	757/122/6	0.853(0.694–1.049)	0.831(0.668–1.034)	1.148(0.433–3.044)
		Negative	1820/360/13	P = 0.131	*P* = 0.098	*P* = 0.781
	rs769450		AA/AG/GG			
		Positive	556/300/29	1.044(0.909–1.2)	1.091(0.927–1.284)	0.831(0.542–1.274)
		Negative	1426/677/90	P = 0.542	*P* = 0.292	*P* = 0.395

*WT* wild type, *HT* heterozygote, *MT* mutant type

^a^: Adjusted for age and sex

Interestingly, in subsequent gender-differentiated analyses, we observed that rs7412-C in APOE was significantly associated with the protection against HEV infection in Chinese Han males (OR < 1.0 adjusted for age, Table 5). However, no significant associations were detected in other three SNPs with HEV infection in the present population.

**Table 5:** Gender-stratification analysis on the association between selected SNPs and anti-HEV IgG seropositivity

Gene nomenclature	SNP	Stratum	Anti-HEV IgG	WT/HT/MT	OR(95%CI)/*P* value^a^		
					Additive	Dominant	Recessive
APOA4	rs1268354			CC/CT/TT			
		Male	positive	187/223/75	0.963(0.823–1.126)	0.893(0.754–1.059)	0.883(0.708–1.101)
			negative	412/587/163	*P* = 0.634	*P* = 0.194	*P* = 0.268
		Female	positive	154/190/54	0.88(0.692–1.117)	1.095(0.812–1.476)	0.833(0.597–1.162)
			negative	366/496/161	*P* = 0.293	*P* = 0.553	*P* = 0.282
APOE	rs405509			TT/TG/GG			
		Male	positive	243/209/32	0.941(0.796–1.112)	1.076(0.894–1.294)	0.998(0.806–1.235)
			negative	581/472/111	*P* = 0.473	*P* = 0.440	*P* = 0.984
		Female	positive	200/168/30	1.089(0.864–1.374)	0.713(0.472–1.075)	1.114(0.714–1.737)
			negative	539/414/71	*P* = 0.471	*P* = 0.106	*P* = 0.635
	rs7412			CC/CT/TT			
		Male	positive	422/63/1	0.707(0.527–0.95)	1.036(0.775–1.385)	0.708(0.522–0.96)
			negative	958/210/6	*P* = 0.021	*P* = 0.811	*P* = 0.026
		Female	positive	335/59/5	0.998(0.728–1.367)	0.351(0.042–2.958)	1.895(0.597–6.014)
			negative	862/159/7	*P* = 0.988	*P* = 0.336	*P* = 0.278
	rs769450			AA/AG/GG			
		Male	positive	297/176/13	1.086(0.9–1.311)	1.004(0.817–1.235)	1.195(0.959–1.49)
			negative	758/355/52	*P* = 0.389	*P* = 0.967	*P* = 0.112
		Female	positive	259/124/16	0.988(0.775–1.26)	0.632(0.34–1.177)	1.116(0.614–2.027)
			negative	668/322/38	*P* = 0.924	*P* = 0.148	*P* = 0.719

*WT* wild type, *HT* heterozygote, *MT* mutant type

^a^ Adjusted for age

## Discussions

In this study, we attempted to evaluate the GWAS-associated SNPs from ABCB1, CYP2E1, CAT, TGFB1 and XRCC1 with HAV infection, and SNPs from APOA4 and APOE with HEV infection in Chinese Han populations, respectively. However, the conflicting results were observed in the present study. First, our results found that rs1045642 C to T variation was associated with the decreased risk of HAV infection in Chinese Han persons, while the results from US study indicated that individuals with T allele of rs1045642 were less prone to be infected by HAV [8]. Second, we observed that rs1001581A to G variation in XRCC1 was significantly associated with protection against HAV infections, whereas this association was not observed in US population. Lastly, Zhang et al. reported that variants in APOA4 and APOE were significantly associated with the lower odds of anti-HEV seropositivity [13]. However, our results only suggested that rs7412 C to T variation in APOE was associated with decreased risks of HEV infection in Chinese Han males.

Several factors could contribute to the differences between our present study and the previous US study. One factor could be ascribed to the genetic heterogeneity of virus. HAV and HEV could be divided into six genotypes (I-VI) and four major genotypes (1–4), respectively [18, 19]. Both HAV and HEV genotypes exhibited a particular geographic distribution [20–22]. Although subgenotype IA HAV constitutes a major fraction of genotype I strains circulating in US and China, the virus strains from the US and China could be further divided into different clusters [23, 24]. In addition, genotype 3 HEV is the main genotype circulating in US, while genotype 4 HEV strain is the dominant genotype in China [25–27]. Thus, the effects of the genetic variation of viruses on the risk of infection in different ethnicities should be investigated in future. Additionally, although the allele frequencies of tested polymorphisms were similar between two studies, the genetic differences among ethnicities might be as the other explanation for the present results (Additional file 3: Table S3) [28, 29]. Therefore, replication studies in different racial/ethnic groups are encouraged.

To our knowledge, this is the first replication study to explore the associations between GWAS-associated SNPs and HAV or HEV infection in China. However, there are several general limitations should be considered. First, although the number of study participants met the requirement for analysis, independent replications in larger population should be conducted in future. Second, our study only sought to replicate the limited SNPs in a Chinese Han population because we felt such a replication would be a critical first step to understanding these results. Greater genome coverage and more informative markers including APOE isoforms are required in order to realize true genetic determinants of hepatitis A and E infections. Last, it is well known that HAV or HEV infections are significantly associated with environmental and social factors. Thus, gene-gene and gene-environment interaction on the risks of virus infection should be further discussed.

## Conclusions

Summary, our present study suggested that ABCB1 and XRCC1 genes variants were significantly associated with the lower risk of HAV infection. Additionally, Chinese Han males with rs7412 C to T variation in APOE are less prone to be infected by HEV. In order to further understand the effects of host genetic factors on HAV or HEV infection in different ethnic populations, more studies using a large-scale association analysis should be warranted in future.

## Additional files

Additional file 1: **Table S1.** Information about primers and probes of selected SNPs. (XLSX 10 kb)

Additional file 2: **Table S2.** Characteristics of selected SNPs. (DOCX 16 kb)

Additional file 3: **Table S3.** Allele frequencies of selected SNPs in Chinese and US populations. (XLSX 13 kb)

## Abbreviations

CI: Confidence intervals
GWAS: Genome-wide association studies
HAV: Hepatitis A virus
HEV: Hepatitis E virus
HWE: Hardy-Weinberg equilibrium
MAF: Minor allele frequency
OR: Odds ratio
SNP: Single-nucleotide polymorphism

## References

[1] Meng QF, You HL, Wang WL, Zhou N, Dong W, Cong W (2015). Seroprevalence and risk factors of hepatitis E virus infection among children in China. J Med Virol.

[2] Wang Z, Chen Y, Xie S, Lv H (2016). Changing epidemiological characteristics of hepatitis A in Zhejiang province, China: increased susceptibility in adults. PLoS One.

[3] Khuroo MS, Khuroo MS (2016). Hepatitis E: an emerging global disease - from discovery towards control and cure. J Viral Hepat.

[4] Vaughan G, Goncalves Rossi LM, Forbi JC, de Paula VS, Purdy MA, Xia G (2014). Hepatitis A virus: host interactions, molecular epidemiology and evolution. Infect Genet Evol.

[5] Zhang P, Li N, Zhu Q, Li F, Yang C, Zeng X (2015). Association between TNFAIP3 nonsynonymous single-nucleotide polymorphism rs2230926 and chronic hepatitis B virus infection in a Chinese Han population. Virol J.

[6] Miao R, Ge H, Xu L, Sun Z, Li C, Wang R (2016). Genetic variants at 18q11.2 and 8q24 identified by genome-wide association studies were not associated with pulmonary tuberculosis risk in Chinese population. Infect Genet Evol.

[7] Rowell JL, Dowling NF, Yu W, Yesupriya A, Zhang L (2012). Trends in population-based studies of human genetics in infectious diseases. PLoS One.

[8] Zhang L, Yesupriya A, Hu DJ, Chang MH, Dowling NF, Ned RM (2012). Variants in ABCB1, TGFB1, and XRCC1 genes and susceptibility to viral hepatitis A infection in Mexican Americans. Hepatology.

[9] Grainger DJ, Heathcote K, Chiano M, Snieder H, Kemp PR, Metcalfe JC (1999). Genetic control of the circulating concentration of transforming growth factor type beta1. Hum Mol Genet.

[10] Kiran M, Saxena R, Chawla YK, Kaur J (2009). Polymorphism of DNA repair gene XRCC1 and hepatitis-related hepatocellular carcinoma risk in Indian population. Mol Cell Biochem.

[11] Yu L, Liu X, Han C, Lu S, Zhu G, Su H (2016). XRCC1 rs25487 genetic variant and TP53 mutation at codon 249 predict clinical outcomes of hepatitis B virus-related hepatocellular carcinoma after hepatectomy: a cohort study for 10 years' follow up. Hepatol Res.

[12] Ros JE, Libbrecht L, Geuken M, Jansen PL, Roskams TA (2003). High expression of MDR1, MRP1, and MRP3 in the hepatic progenitor cell compartment and hepatocytes in severe human liver disease. J Pathol.

[13] Zhang L, Yesupriya A, Chang MH, Teshale E, Teo CG (2015). Apolipoprotein E and protection against hepatitis E viral infection in American non-Hispanic blacks. Hepatology.

[14] Mahley RW, Rall SC (2000). Apolipoprotein E: far more than a lipid transport protein. Annu Rev Genomics Hum Genet.

[15] Mahley RW (1988). Apolipoprotein E: cholesterol transport protein with expanding role in cell biology. Science.

[16] Lu J, Zhou Y, Lin X, Jiang Y, Tian R, Zhang Y (2009). General epidemiological parameters of viral hepatitis A, B, C, and E in six regions of China: a cross-sectional study in 2007. PLoS One.

[17] Yu P, Huang L, Li H, Liu M, Zong J, Li C, et al. Epidemiological investigation of an outbreak of hepatitis A in rural China. Int J Infect Dis. 2015;33:191–5. 10.1016/j.ijid.2015.02.006 25677727

[18] Cristina J, Costa-Mattioli M (2007). Genetic variability and molecular evolution of hepatitis A virus. Virus Res.

[19] Sridhar S, Lau SK, Woo PC (2015). Hepatitis E: a disease of reemerging importance. J Formos Med Assoc.

[20] Blum HE (2016). History and global burden of viral hepatitis. Dig Dis.

[21] Pérez-Gracia MT, García M, Suay B, Mateos-Lindemann ML (2015). Current knowledge on hepatitis E. J Clin Transl Hepatol.

[22] Wang H, Wang XY, Zheng HH, Cao JY, Zhou WT, Bi SL (2015). Evolution and genetic characterization of hepatitis A virus isolates in China. Int J Infect Dis.

[23] Klevens RM, Miller JT, Iqbal K, Thomas A, Rizzo EM, Hanson H (2010). The evolving epidemiology of hepatitis a in the United States: incidence and molecular epidemiology from population-based surveillance, 2005-2007. Arch Intern Med.

[24] Murphy TV, Denniston MM, Hill HA, McDonald M, Klevens MR, Elam-Evans LD (2016). Progress toward eliminating hepatitis A disease in the United States. MMWR Suppl.

[25] Ren F, Zhao C, Wang L, Wang Z, Gong X, Song M (2014). Hepatitis E virus seroprevalence and molecular study among blood donors in China. Transfusion.

[26] Dalton HR, Webb GW, Norton BC, Woolson KL, et al. Hepatitis E virus: time to change the textbooks. Dig Dis. 2016;34:308–16. 10.1159/000444468 27170383

[27] Kuniholm MH, Purcell RH, McQuillan GM, Engle RE, Wasley A, Nelson KE (2009). Epidemiology of hepatitis E virus in the United States: results from the third National Health and nutrition examination survey, 1988-1994. J Infect Dis.

[28] Huang N, Agrawal V, Giacomini KM, Miller WL (2008). Genetics of P450 oxidoreductase: sequence variation in 842 individuals of four ethnicities and activities of 15 missense mutations. Proc Natl Acad Sci U S A.

[29] Kambhampati A, Payne DC, Costantini V, Lopman BA (2016). Host genetic susceptibility to enteric viruses: a systematic review and metaanalysis. Clin Infect Dis.

