# Supplementary material for: Evaluation of candidate genes associated with hepatitis A and E virus infection in Chinese Han population
Source: Virol J. 2018 Mar 20;15:47. doi: 10.1186/s12985-018-0962-2 (PMC5859746; doi:10.1186/s12985-018-0962-2)
Supplement: Supplementary file 2 — Table S2. Characteristics of selected SNPs. (DOCX 16 kb) [file 12985_2018_962_MOESM2_ESM.docx]

Supplement table 2 Characteristics of selected single-nucleotide polymorphisms

| rs | Chromosome | Position | Allele | TFBS/Splicing(ESE or ESS) | Nearby Gene | Effect Allele | minor |
| --- | --- | --- | --- | --- | --- | --- | --- |
| rs769214 | 11 | 34416293 | G/A | Y | ABTB2\|\|CAT | 0.287 | A |
| rs1001581 | 19 | 48757228 | A/G | -- | XRCC1 | 0.44 | G |
| rs1045642 | 7 | 86976581 | C/T | Y | ABCB1 | 0.402 | T |
| rs1800469 | 19 | 46552136 | C/T | Y | TGFB1\|\|B9D2 | 0.47 | T |
| rs2031920 | 10 | 1.35E+08 | C/T | Y | OR7M1P\|\|CYP2E1 | 0.265 | T |
| rs1268354 | 11 | 1.16E+08 | C/T | -- | APOA5\|\|APOA4 | 0.417 | T |
| rs405509 | 19 | 50100676 | T/G | Y | TOMM40\|\|APOE | 0.31 | G |
| rs7412 | 19 | 50103919 | C/T | -- | APOE/LOC100129500 | 0.108 | T |
| rs769450 | 19 | 50102284 | A/G | -- | APOE | 0.196 | G |
